# Effect of Anti-TNF Therapy on Mucosal Apoptosis Genes Expression in Crohn's Disease

**DOI:** 10.3389/fimmu.2021.615539

**Published:** 2021-03-09

**Authors:** Liliana Lykowska-Szuber, Michal Walczak, Marzena Skrzypczak-Zielinska, Joanna Suszynska-Zajczyk, Kamila Stawczyk-Eder, Katarzyna Waszak, Piotr Eder, Anna Wozniak, Iwona Krela-Kazmierczak, Ryszard Slomski, Agnieszka Dobrowolska

**Affiliations:** ^1^Department of Gastroenterology, Dietetics and Internal Diseases, Poznan University of Medical Sciences, Poznan, Poland; ^2^Institute of Human Genetics, Polish Academy of Sciences, Poznan, Poland; ^3^Department of Biochemistry and Biotechnology, Poznan University of Life Sciences, Poznan, Poland; ^4^The NanoBioMedical Centre, Adam Mickiewicz University, Poznan, Poland

**Keywords:** Crohn's disease, anti-TNF therapy, immunomodulation, genes expression, apoptosis

## Abstract

Crohn's disease (CD) is a chronic immune-mediated disorder for which there is not a fully effective treatment. Moreover, biological therapy with anti-tumor necrosis factor-α (anti-TNF-α) monoclonal antibodies leads to an effective response in only 60–70% of patients. Our previous data suggested that specific *loci* polymorphism of the *TNFRSF1B, FCGR3A, IL1R, IL1B*, and *FAS* genes could be a predictor of the primary non-response to anti-TNF therapy in CD patients. In this work, we propose to explain this hypothesis by functional analysis in colon biopsies and in a cell culture model. Using the RT-qPCR analysis, we estimated the *FCGR3A, IL1R, TNFRSF1B, IL1B, FAS*, and *ADAM17* genes mRNA level in colon biopsies material from inflamed and non-inflamed tissue from 21 CD patients (14 responders and 7 non-responders to anti-TNF therapy) and 6 controls, as well as *in vitro* in a peripheral blood mononuclear cells (PBMCs) from 14 CD patients (seven responders and seven non-responders to anti-TNF therapy) and eight controls cultured for 72 h with 10 μg/ml of anti-TNF antibody. Our findings demonstrated a significant down-regulation of *TNFRSF1B* gene expression in non-responders both in inflamed and in non-inflamed colon tissue, while the expression of the *FCGR3A* and *IL1B* genes was significantly up-regulated in non-responders in the inflamed colon region. *In vitro* research results indicate that the anti-TNF drug induced a significant decrease in *TNFRSF1B, FCGR3A*, and *FAS* gene expression in non-responders. These results show that altered *TNFRSF1B, FCGR3A*, and *IL1B* genes expression can be a predictor of the primary non-response to anti-TNF therapy in CD patients.

## Introduction

Crohn's disease (CD) is a type of inflammatory bowel disease (IBD), which generally affects the ileum and colon and is the result of an abnormal inflammatory response to antigens derived from the gastrointestinal tract (1). The main cytokine with a proven role in the pathogenesis of CD is the tumor necrosis factor TNF alpha (TNF-α). Its increased expression is found in both intestinal inflammatory infiltrates and in the serum of patients (2). In the last few years, the therapy that involves the blockage of this cytokine has been increasingly used in the treatment of IBD (3). With the use of anti-TNF-α drugs, it is possible to achieve clinical and endoscopic remission of the disease. However, there is a group of about one-third of IBD patients who primarily do not respond to this type of therapy. Despite the anti-TNF-α treatment, no clinical improvement or inhibition of the inflammatory process in the endoscopic image is observed (4, 5).

While there are many pro-inflammatory cytokines in the inflamed mucosa, TNF-α is one of the main cytokines. CD is characterized by high activity of Th1/Th17 lymphocytes, which produce pro-inflammatory cytokines, such as interferon-gamma, interleukin 17A and interleukin 2. The increase in the expression of pro-inflammatory cytokine genes thus reflects the degree of activity of the immune system involved in the pathogenesis of the disease. Biological therapy induces endoscopic remission, which should be reflected by the reduction of inflammatory activity in the intestinal mucosa (6). On this basis, it can be hypothesized that a complete response to treatment is manifested by a reduction in the activity of pro-inflammatory cytokines in the intestinal mucosa and the associated decrease in the expression of genes encoding individual cytokines, as well as other proteins associated with the onset of and decrease in the inflammatory process. The exact mechanism of action of anti-TNF antibodies in IBD is still uncertain, but there is a general consensus that one of their main therapeutic properties is mediated by binding to membrane-bound TNF (mTNF)-bearing immune cells in intestine mucosa. These drugs bind to mTNF-expressing macrophages, thereby inducing apoptosis in TNF-RII-expressing mucosal T cells. The mTNF/TNF-RII signaling pathway is therefore a basis regulator in mediating resistance to intestinal T-cell apoptosis and may contribute to the perpetuation of mucosal inflammation (7–9). Moreover, our previous data suggested that specific *loci* of apoptosis genes including TNF receptor superfamily member 1B (*TNFRSF1B*, OMIM 191191), Fc fragment of IgG receptor IIIa (*FCGR3A*, OMIM 146740), interleukin 1 receptor type 1 (*IL1R*, OMIM 147810), interleukin 1 beta (*IL1B*, OMIM 147720) and Fas cell surface death receptor (*FAS*, OMIM 134637) genes could be predictors of the primary non-response to anti-TNF therapy in CD patients (10). In this work, we propose to explain this hypothesis by functional analysis based on the real-time quantitative PCR (RT-qPCR) in the colon biopsies of CD patients treated with anti-TNF and in cell culture model.

## Materials and Methods

### Clinical Characterization

All patients enrolled in this study were hospitalized in the Department of Gastroenterology, Dietetics, and Internal Diseases of Poznan University of Medical Sciences in Poznan, Poland. They were assigned to two groups. The first group consisted of 21 individuals, for whom the research was carried out on tissue material collected during a routine colonoscopy. The second group included 13 patients, from whom peripheral blood samples for cell culture studies were collected. The detailed clinical characteristics of both study groups are presented in Tables 1, 2. All patients had a confirmed diagnosis of CD. The subjects were treated with anti-TNF monoclonal antibodies (mAb') in the therapeutic program of the National Health Fund (which is an official reimbursement program for all biological therapies in Poland) in the Department of Gastroenterology, Dietetics, and Internal Diseases of Poznan University of Medical Sciences in Poznan between 2013 and 2019. The inclusion criteria for patients were that they had to be aged >18 years, have a diagnosis of active CD, be biologic-naïve, as well as having had treatment failure or intolerance to first-line therapies, such corticosteroids, and/or immunosuppressants. The exclusion criteria were the presence of an ileostomy or colostomy and infectious complications (including intraabdominal infections). The diagnosis was based on previously defined criteria (12). Clinical disease activity was assessed by using the Crohn's Disease Activity Index (CDAI) (13). Patients who had never smoked or had quit smoking at least 10 years prior to participating in the study were considered non-smokers. The patients were administered infusions of infliximab (IFX) at a dose of 5 mg/kg body weight at Weeks 0, 2, 6 (the induction phase), and then every 8 weeks until 1 year (54 weeks-the maintenance phase). Adalimumab (ADA) was given subcutaneously at Week 0 at a dose of 160 mg, 80 mg was given at Week 2, and then every other week a 40-mg dose was given until 1 year (54 weeks).

**Table 1:** Characteristics of the patients group in the mucosa studies.

**Parameter**	**Total patients *n* = 21**	**Responders *n* = 14**	**Non-responders *n* = 7**	***p*-value**
Gender, (F/M), *n* (%)	10 (47.61)/11 (52.39)	7 (50.00)/7 (50.00)	3 (42.86)/4 (57.14)	0.7574
Age, (years) mean ± SD	33.81 ± 12.51	30.64 ± 8.21	40.14 ± 17.47	0.2858
Smoker, *n* (%)	1 (4.76)	1 (7.14)	0 (0.00)	0.4687
Previous surgeries, *n* (%)	8 (38.09)	2 (14.28)	6 (85.71)	0.0015
Disease duration, months, mean ± SD	59.62 ± 42.42	53.29 ± 35.51	72.29 ± 54.63	0.5190
Intestinal location,^*^n **(%)**				
Colonic (L2)	6 (28.57)	3 (21.43)	3 (42.86)	0.3055
Ileal (L1)	4 (19.05)	3 (21.43)	1 (14.28)	0.6944
Ileocolonic (L3)	11 (52.38)	8 (57.14)	3 (42.86)	0.5366
**Behavior**, ***n*** **(%)**				
Nonstricturing nonpenetrating (B1)	21 (100.00)	14 (100.00)	7 (100.00)	>0.9999
Stricturing (B2)	0 (0.00)	0 (0.00)	0 (0.00)	>0.9999
Penetrating (B3)	0 (0.00)	0 (0.00)	0 (0.00)	>0.9999
Histological score, median (range)	10 (6–14)	9 (6–14)	12 (8–14)	0.0744
**Medication**, ***n*** **(%)**				
Mesalamine	21 (100.00)	14 (100.00)	7 (100.00)	>0.9999
Corticosteroids	8 (38.90)	4 (28.57)	4 (57.14)	0.2037
Azathioprine	13 (61.90)	9 (63.28)	4 (57.14)	0.7507
Adalimumab	4 (19.05)	3 (21.43)	1 (14.28)	0.6944
Infliximab	17 (80.95)	11 (78.57)	6 (85.71)	0.6944

* *Disease locations were classified according to the Montreal Classification (11)*.

**Table 2:** Characteristics of patients group in PBMC culture studies.

**Parameter**	**Total patients *n* = 14**	**Responders *n* = 7**	**Non-responders *n* = 7**	***p*-value**
Gender, F/M, *n* (%)	4 (28.57)/10 (71.43)	2 (28.57)/5 (71.43) 33.33/66.67 (%)	2 (28.57)/5 (71.43)	>0.9999
Age, (years) mean ± SD	29.86 ± 7.29	28.71 ± 6.08	31 ± 8.68	0.7372
Smoker, *n* (%)	0 (0.00)	0 (0.00)	0 (0.00)	>0.9999
Previous operations, *n* (%)	6 (42.86)	2 (28.57)	4 (57.14)	0.2801
Disease duration, months, mean ± SD	61.71 ± 50.44	65.14 ± 47.93	58.29 ± 56.47	0.5233
Intestinal location,^*^n **(%)**				
Colonic (L2)	4 (28.57)	2 (28.57)	2 (28.57)	>0.9999
Ileal (L1)	2 (14.29)	2 (28.57)	0 (0.00)	0.1266
Ileocolonic (L3)	7 (50.00)	3 (42.86)	5 (71.43)	0.2801
**Behavior**, ***n*** **(%)**				
Non-stricturing, non-penetrating (B1)	10 (71.43)	6 (85.71)	4 (57.14)	0.2367
Stricturing (B2)	2 (14.29)	1 (14.28)	1 (14.28)	>0.9999
Penetrating (B3)	2 (14.29)	0	2 (28.57)	0.1266
**Medication**, ***n*** **(%)**				
Mesalamine	14 (100.00)	7 (100.00)	7 (100.00)	>0.9999
Corticosteroids	4 (28.57)	1 (14.28)	3 (42.86)	0.2367
Azathioprine	8 (57.14)	3 (42.86)	5 (71.43)	0.2801
Adalimumab	6 (42.86)	3 (42.86)	3 (42.86)	>0.9999
Infliximab	7 (50.00)	4 (57.14)	3 (42.86)	0.5930

* *Disease locations were classified according to the Montreal Classification (11)*.

**Figure 1:**
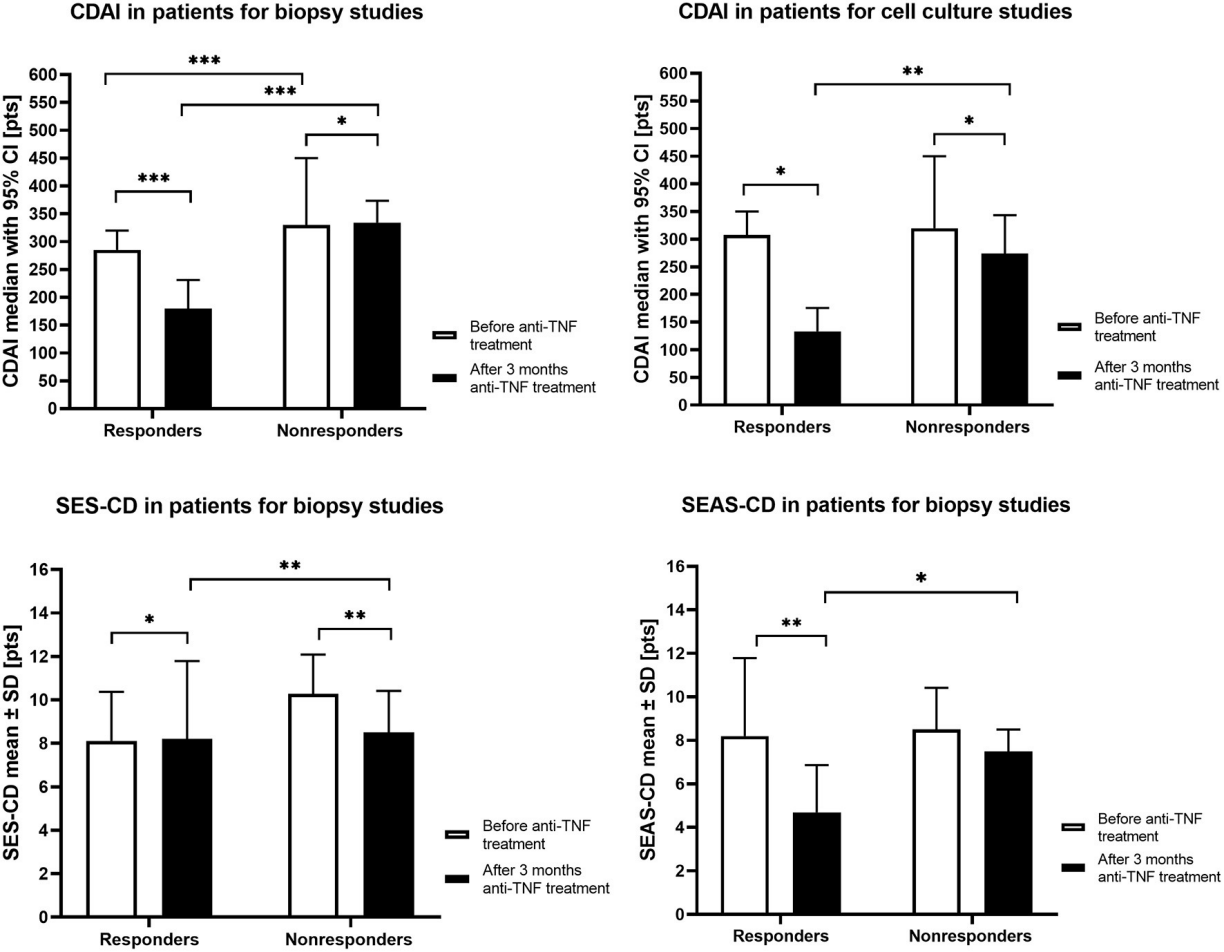
The clinical parameters in responders and non-responders CD patients groups before anti-TNF treatment and after 3 months of therapy. CDAI, Crohn's Disease Activity Index; SES-CD, Simple Endoscopic Score for Crohn's Disease; SEAS-CD, Simple Enterographic Activity Score for Crohn's Disease; CI, confidence interval; SD, standard deviation; *p < 0.05, **p < 0.01, ***p < 0.001.

The anti-TNF treatment response was assessed following 12 weeks of the therapy. The CDAI score was used to determine the clinical response. The clinical response to the therapy was defined as a CDAI reduction by ≥70 points. Moreover, each patient in the mucosal studies group, before qualifying for treatment and after 12 weeks of therapy, had a colonoscopy and/or, in the case of small intestine involvement, MR enterography. The activity of lesions in colonoscopy was assessed according to the Simple Endoscopic Score for Crohn's Disease (SES-CD) and the Simple Enterographic Activity Score for Crohn's Disease (SEAS-CD) in MR enterography (14, 15) (Figure 1).

The control groups consisted of 6 healthy (two female, four male) subjects with an average age of 46.7 years in mucosal studies and eight healthy subjects (four female, four male) with an average age 40.2 years in PBMCs culture studies.

All individuals gave their written consent to genetic testing and the evaluation of biochemical parameters in serum and colonoscopy examination. The research was approved by the Bioethical Committee of Poznan University of Medical Sciences, Poland, under Resolution No. 762/13 approved on 9 November 2013 and Resolution No. 1042/18 approved on 11 October 2018. All experiments were performed in accordance with the principles of the 1964 Declaration of Helsinki with its later amendments.

### Biopsy Preparation

Approximately 1–2 mg of biopsies were obtained from sites of inflammation and non-inflamed regions from treatment-naïve patients with CD and from healthy controls during a colonoscopy. Next, the collected biopsies were suspended in 300 μl of RNALatter® reagent (Sigma) and were frozen at −80°C until RNA isolation begun started. In each case, material for microscopy analysis was also collected (H+E). We assessed the collected material according to the CD histopathological activity. The applied index was developed by D'Haens et al. and it consists of 8 histological parameters (variables) assessed individually (16). The results are presented in Table 1.

### PBMCs Isolation, Culture, and Treatment With the Antibody

PBMCs were isolated from 9 ml samples of whole blood using LYMPHOSEP™ (MP Biomedicals LLC, Ohio, USA), according to the manufacturer's instructions. The obtained pellet was suspended in 4 ml of a Lymphogrow medium (Cytogen-Polska Sp. z o.o., Zgierz, Poland) containing phytohemagglutinin (PHA) and recombinant IL-2 (4 ng, 100 U, BioLegend, San Diego, CA, USA). The suspension was then transferred to a 25-ml vessel for adherent culture. Cells were grown under standard conditions at 37°C, 5% CO_2_ with shaking for 24 h. Non-adherent cells were washed with PBS and transferred to a 25-ml vessel for suspension culture with fresh Lymphogrow medium supplemented with IL-2. After another 48 h, the cells were passaged and maintained in a culture using a standard RPMI-1630 medium supplemented with L-glutamine (2 mM), FBS (10%), penicillin (100 IU/ml), streptomycin (100 μg/ml), and with the addition of IL-2. Cell differentiation was measured by CD3, CD4, CD8, CD45, and HLA-DR by flow cytometry analysis. In the third passage, anti-TNF mAbs' (Sigma) was added (10 μg/ml). In parallel, a control culture without the addition of the antibody was carried out. After 72 h of culture, cells were suspended in 200 μl stayRNA solution (A&A Biotechnology, Gdansk, Poland) and frozen at −80°C for RNA isolation. Moreover, part of the cells was subjected to apoptosis analysis.

### Apoptosis Assessment

Apoptosis was measured using a Muse® Annexin V and Dead Cell Assay Kit (Merck, Darmstadt, Germany) in a Muse Cell Analyzer (Luminex Corporation, Austion, USA), according to manufacturer's protocol. The cells were resuspended in a medium containing at least 1% FBS. Hundred microliter of Muse™ Annexin V & Dead Cell Reagent and 100 μL of cells were added to each tube. The cell suspension was incubated (RT, 20 min) and loaded onto a Muse® Cell Analyzer.

### RNA Isolation, cDNA Synthesis, and Quality Control

Tissue samples suspended in TRI Reagent® (Sigma-Aldrich, Saint Louis, MO, USA) were homogenized with mortar and pestle and subjected to RNA isolation with RNeasy Mini Kit (Qiagen, Hilden, Germany). The total RNA from cell culture containing up to 10^5^-5 × 10^5^ cells was extracted using Total RNA Mini Plus Concentrator (A&A BIOTECHNOLOGY, Gdansk, Poland), according to the manufacturer's procedure. For all the RNA samples obtained, a quantitative and qualitative evaluation was carried out using Agilent RNA 6000 Nano Kit and the Bioanalyzer 2.0 equipment (Agilent, Santa Clara, CA, USA). A 900 ng of total RNA with RIN ≥ 7 was converted to cDNA with an iScript Advanced Reverse Reaction kit (Bio-Rad) with the following conditions: 25°C for 5 min – annealing step, 42°C for 30 min – reverse transcription and 95°C for 1 min – inactivation.

### Real-Time Quantitative PCR (RT-qPCR)

The mRNA level of selected genes: *ADAM17, FAS, FCGR3A, IL1B, IL1R*, and *TNFRSF1B* was measured by a real-time quantitative polymerase chain reaction on a BioRad CFX Connect 96-well Thermal Cycler (Bio-Rad Laboratories, Inc., Hercules, California, United States) using the iTaq UniverSYBR Green assay (Bio-Rad Laboratories, Inc., Hercules, California, USA), according to the manufacturer's instructions. Primers for *ADAM17, FAS, FCGR3A, IL1B*, and *IL1R* genes analysis were designed using a Primer-BLAST tool, and their sequences are presented in Table 3. Primers for *TNFRSF1B* gene, as well as reference *PPIA* and *RPLP0* genes, were ordered as PrimePCR^TM^ SYBR® Green Assay by Bio-Rad Laboratories, Inc. manufacturer. The results of qPCR reactions are presented as dCt = (dCt_referencegene_ – dCt_geneofinterest_). Every reaction was performed in duplicate.

**Table 3:** Primers sequence and qPCR parameters.

**Gene**	**Sequence 5**′**-3**′****	**Annealing temperature**	**Amplicon (bp)**	**Reaction efficiency (%)**
*ADAM17*	AGAATGTTTCACGTTTGCAGTCTC	55–65°C	117	97.95
	CTCGATGAACAAGCTCTTCAGGTG			
*FAS*	GTGAGGGAAGCGGTTTACGA	55–65°C	193	96.61
	AGATGCCCAGCATGGTTGTT			
*FCGR3A*	CACATATTTACAGAATGGCACAGG	55–65°C	173	95.31
	ACACTGCCAAACCTTGAGTGATGG			
*IL1B*	AAAGCTTGGTGATGTCTGGTC	55–65°C	89	91.62
	GGACATGGAGAACACCACTTG			
*IL1R*	TTGGGTTAAGAGGACAGGGA	55–65°C	105	90.00
	TGATTTCTTCTCTGGAGGCTG			

### Statistical Analysis

Analysis of gene expression in intestinal biopsies was based on a multiple comparison non-parametric Kruskal-Wallis test (due to the failure to meet the assumptions for parametric tests) which compare every group in terms of the median value. *Post-hoc* analysis was performed based on Dunn's test, taking into account the Benjamin-Hochberg correction for multiple comparisons to specify which groups are significantly different. The Mann-Whitney non-parametric test was used to compare median value of the two groups in case of apoptosis and gene expression analysis in T lymphocytes cultures, as well as to compare clinical parameters between responders and non-responders. *P*-values < 0.05 were considered as statistically significant. All analyses were performed using R software (version 3.2.3) and R Studio.

## Results

Previously, in the research concerning the identification of molecular markers useful in predicting non-response to anti-TNF biological agents, we described that polymorphism in apoptosis and inflammatory pathways genes could be associated with this phenomenon in CD patients (10). These investigations were performed based on long-range PCR libraries and next-generation sequencing analysis of the selected genes panel (17). In the present study, we test whether changes in mRNA expression of these genes are associated with a primary non-response in our group of Polish patients. We also investigate the impact of anti-TNF mAbs on patients' T cells apoptosis and how the treatment affects the activity of selected targets.

### Gene Expression in Intestinal Biopsy Samples

**Figure 2:**
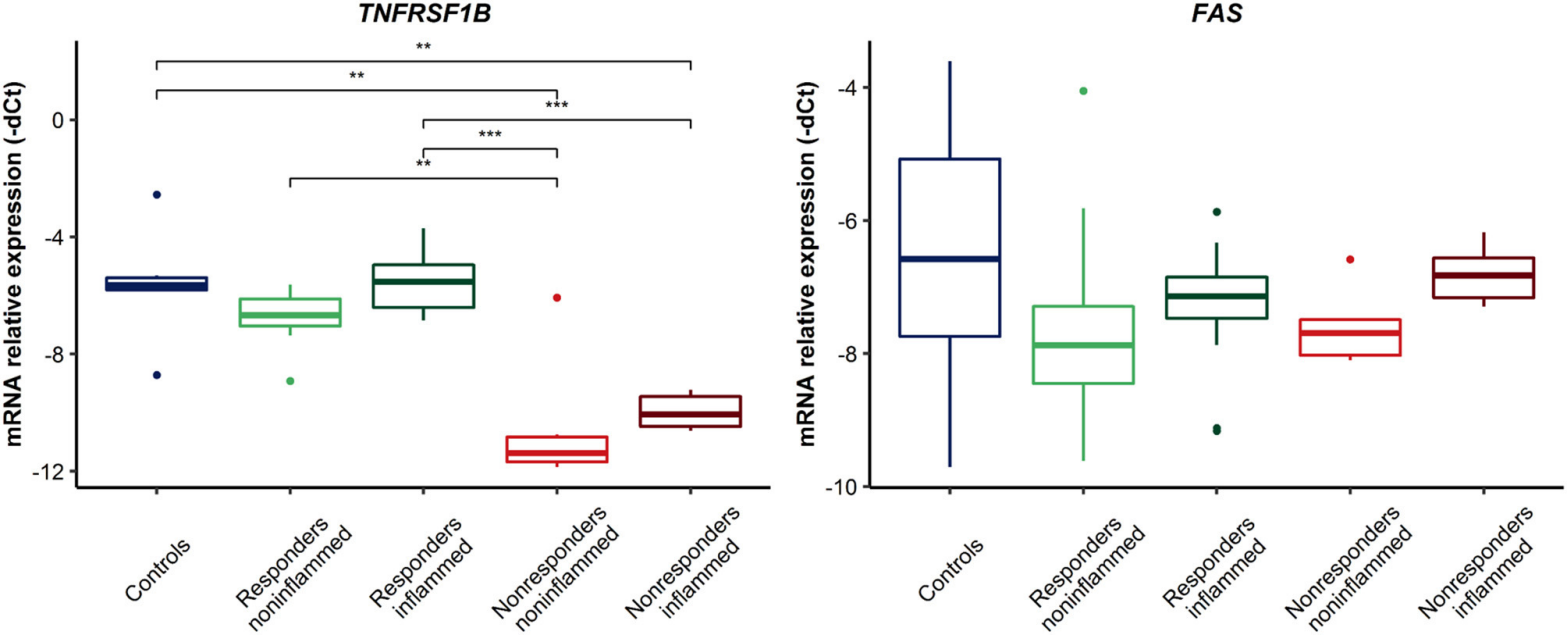
Relative expression of genes involved in apoptosis in CD patients' colon biopsies. ***p < 0.001; **p < 0.01.

**Figure 3:**
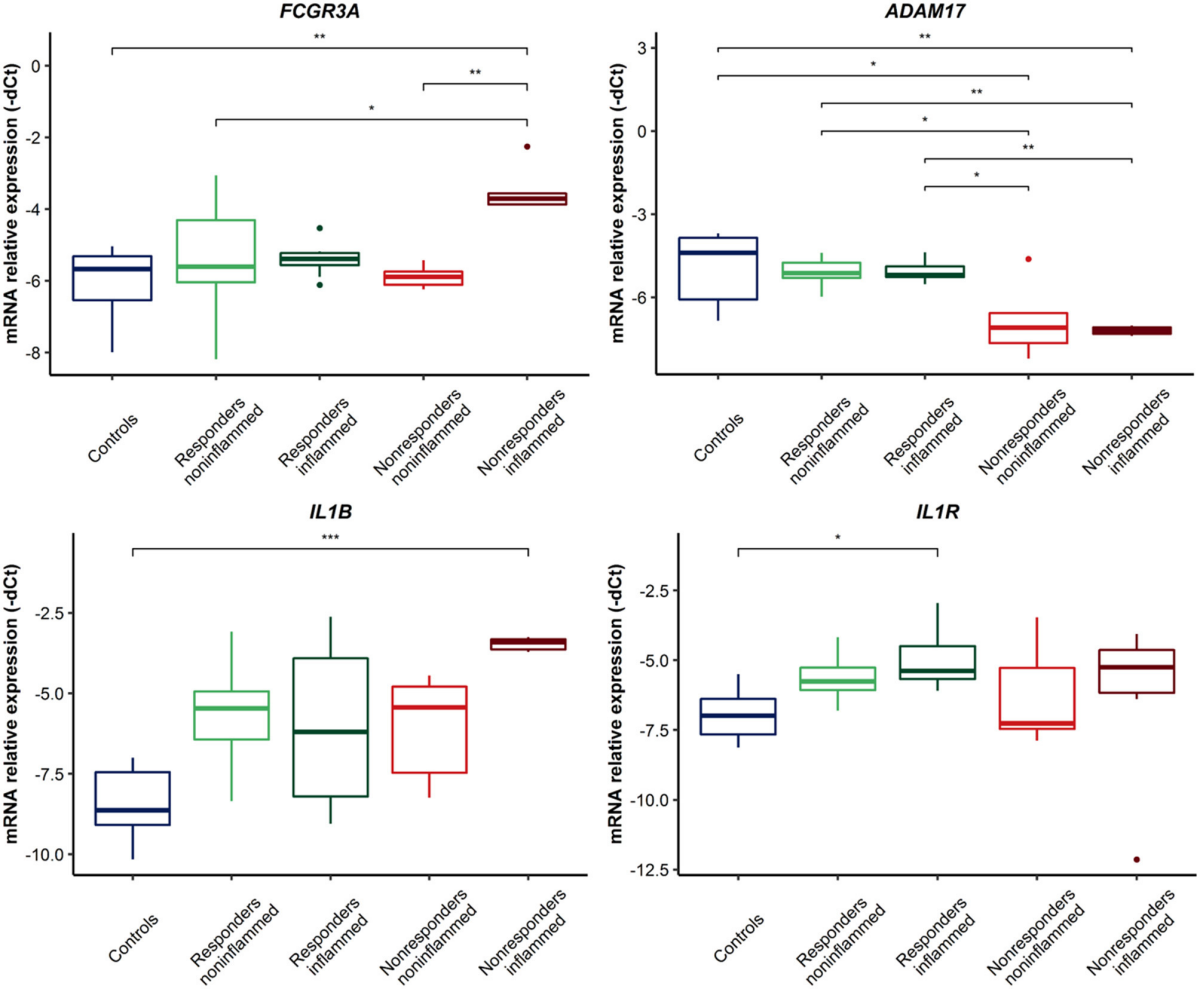
Relative expression of genes involved in TNFα and anti-TNF monoclonal antibodies action in colon biopsies. ***p < 0.001; **p < 0.01; *p < 0.05.

The mRNA expression level of *TNFRSF1B, FAS, FCGR3A, IL1B, IL1R*, and *ADAM17* genes was investigated to find potential differences between patients who did not respond to the therapy, patients who did respond, and healthy individuals. Additionally, we compared the expression in the inflamed and non-inflamed regions. In addition to the *FAS* gene, this analysis demonstrated significant differences in the mRNA level between the above-mentioned groups. For *TNFRSF1B*, in non-responders, both the inflamed and non-inflamed tissue presented a decreased level of the gene compared to biopsies from the responders and control group (Figure 2) (detailed values shown in Supplementary Table 1). *FAS* and *TNFRSF1B* genes are essential mediators in apoptosis pathways. Furthermore, other molecular factors involved in TNFα biology were investigated. For the *ADAM17* gene, a decreased level of transcripts was demonstrated in the material from non-responders to anti-TNF in inflamed and non-inflamed biopsies (Figure 3, Supplementary Table 2), similar to *TNFRSF1B*. On the contrary, the *FCGR3A* gene showed up-regulated levels in non-responding patients, though only in inflamed tissues when compared to the controls (*p* = 0.0044, Figure 2). Moreover, it should be emphasized that a relevant difference was found in non-responding patients between non-inflamed and inflamed colon tissue (*p* = 0.0033, Figure 3). Likewise, in the case of *IL1B* and its receptors, some significant differences were observed. The *IL1B* mRNA level was up-regulated in inflamed tissues on non-responders as compared to the control group (*p* = 0.0002, Figure 3). For the *IL1R* gene, there was only a significant difference between the control group and inflamed biopsy from responders (*p* = 0.0047, Figure 3).

### Infliximab Did Not Induce Apoptosis in T Lymphocytes

PBMC fractions of patients and controls after stimulation of the cell culture with interleukin 2 in order to increase the fraction required for cell research and confirm the positive stimulation were subjected to a flow cytometry analysis to assess the differentiation level of the cells. On average CD3^+^ lymphocytes represented 74.8% (SD ± 30.3%) of the whole fraction. CD3^+^ CD4^+^ - 31.3% (SD ± 16.6%), CD3^+^ CD8^+^ - 31.3% (SD ± 13.5%) and NK cells represented 9.4% (SD ± 4.5%). Based on the available data and the cytotoxicity test (data not shown), it should be assumed that 0–100 μg/ml of infliximab is the concentration *in vitro*, which reflects the concentration of the drug in plasma (18, 19).

The impact of anti-TNF mAbs on apoptosis of T lymphocytes was investigated by incubation with 10 μg/ml of infliximab for 72 h with cells obtained from responders, non-responders, and healthy controls. Simultaneously, control cultures were grown. Then, the overall apoptosis level was measured. However, no statistically significant differences between any of the three groups and parallel controls were observed. The cells viability was on average 74.9% (SD ± 8.8), 69.9% (SD ±12.1), and 72.7% (SD ± 7.5) for controls, responders and non-responders treated with anti-TNF vs. 75.4% (SD ± 10.4), 71.7% (SD ±10.5), and 73.1% (SD ±5.7) for untreated cells, respectively.

### Infliximab Modified Expression Level of Apoptosis-Related Genes

**Figure 4:**
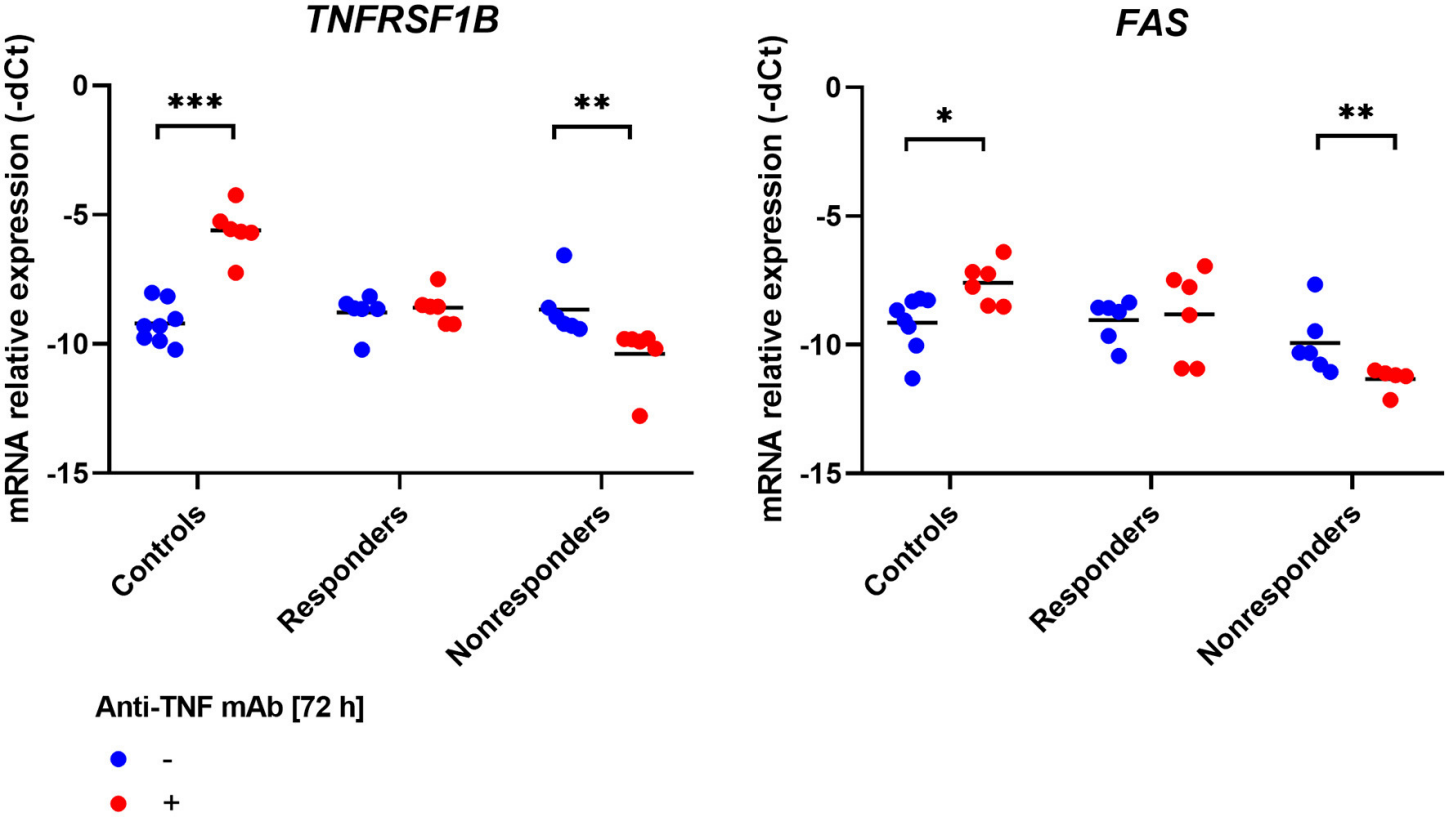
Relative expression of receptor genes involved in apoptosis in PBMCs culture treated with anti-TNF monoclonal antibodies. ***p < 0.001; **p < 0.01; *p < 0.05.

**Figure 5:**
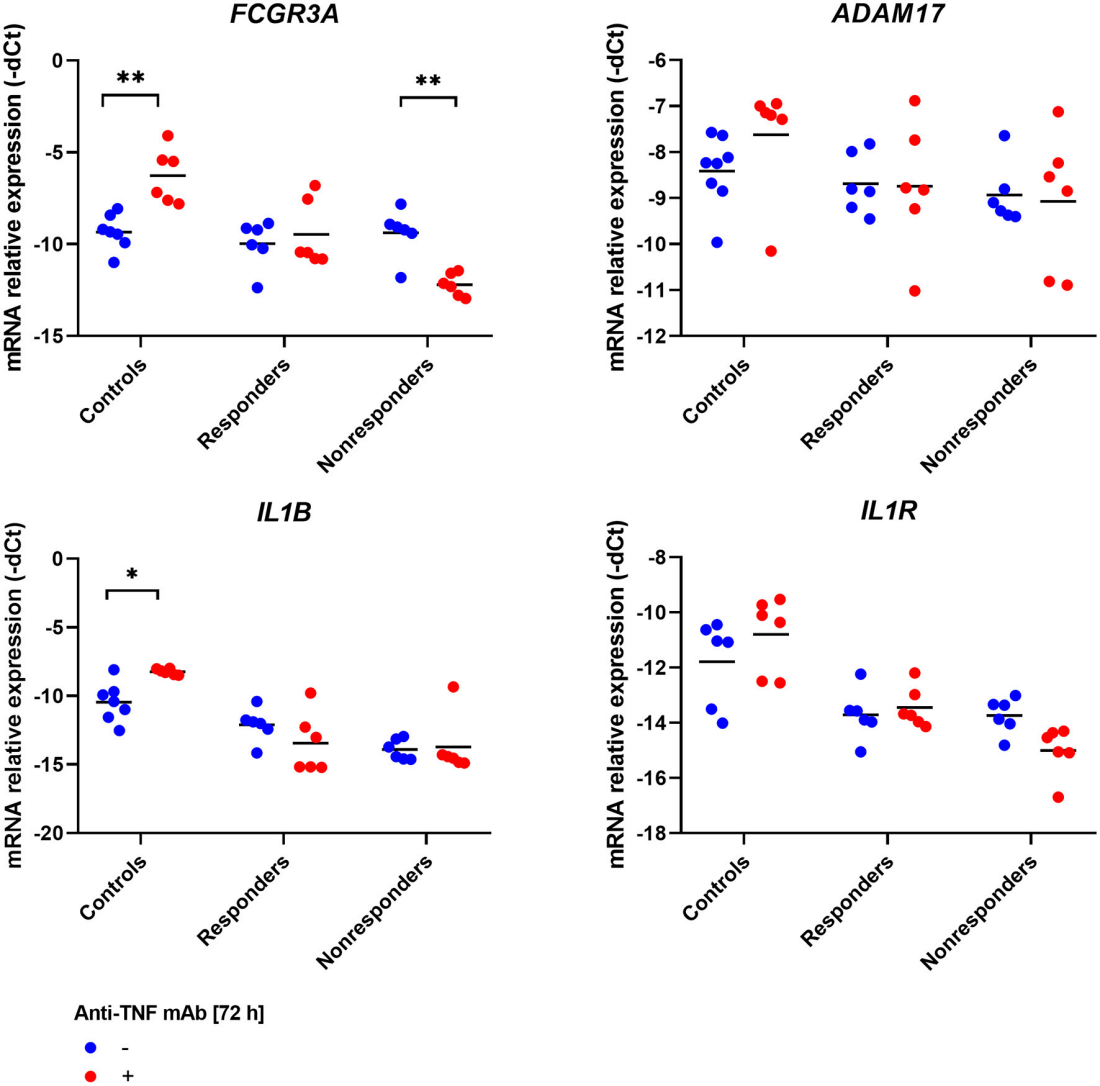
Relative expression of receptor genes involved in the action of TNFα and anti-TNF monoclonal antibodies in PBMCs culture treated with anti-TNF drug. **p < 0.01; *p < 0.05.

However, no impact of infliximab on T cells apoptosis was observed. We wondered whether the treatment can modify the expression level of our genes of interest. Indeed, in the case of two apoptotic genes – *TNFRSF1B* and *FAS*, and interestingly also in *FCGR3A*, which is responsible for antibody-independent cellular cytotoxicity (AICC) mechanism, the same pattern of changes in expression was described. In healthy controls, the significant induction of mRNA expression was observed between treatment and control (*TNFRSF1B, p* = 0.0007; *FAS, p* = 0.01998; *FCGR3A, p* = 0.0012). In the case of responding patients, no significant changes were detected, and in non-responding patients, down-regulation of the three genes occurred (*TNFRSF1B, p* = 0.0022; *FAS, p* = 0.0087; *FCGR3A, p* = 0.0087; Figures 4, 5).

For the remaining genes, only in *IL1B* mRNA in the control group was a significant increment observed (*p* = 0.0217, Figure 5).

## Discussion

The introduction of anti-TNF agents in CD therapy was an irrefutable breakthrough, although this form of treatment is not effective in almost 30–40% of patients. *In vivo*, inflammatory processes are complex and multifactorial, and their exact mechanism is still a matter of debate. In previous studies, based on a custom panel of genes and NGS technology, the genetic background of non-response was investigated in a selected group of genes which seem to play a key role in TNFα biology and anti-TNF action. Polymorphic variants of *TNFRSF1B, FAS, FCGR3A, IL1B*, and *IL1R*, genes were identified as potentially associated with a lack of response to mAbs' (10). However, mostly intronic, 3'-UTR location suggests their potential impact on gene expression regulation. Since genes that remain altered in non-responders constitute a potential new therapeutic target to induce remission in anti-TNF refractory patients, their activity was the subject of this work. Moreover, the *ADAM17* gene, which encodes the main enzyme responsible for converting transmembrane TNF-α into its soluble form, was included in our research. Thus, in intestinal biopsies, significant changes in mRNA expression levels were detected between responders, non-responders, and the control group. Down-regulation of *TNFRSF1B* and *ADAM17* mRNA was detected in inflamed and non-inflamed tissues of refractory patients. Additionally, in non-inflamed biopsies of non-responders, a significant increment of *FCGR3A* and *IL1B* was described. These expression changes seem to be important and could play a direct role in mAbs action. To provide the answer as to whether mAbs influences the mRNA activity of the genes in question, we performed an experiment on T lymphocytes treated with the anti-TNF drug. Overall, no influence of anti-TNF on cell apoptosis was observed, although somehow the treatment modified significantly the expression of *TNFRSF1B, FAS*, and *FCGR3A* in the same pattern, up-regulating mRNA level in the control group and down-regulating in non-responders, with no significant effect in patients who responded to anti-TNF mAbs. However, it should be noted that the lack of differences in cell viability measured by a flow cytometry does not exclude the initiation of apoptosis-related processes manifested by a change in concentration of membrane proteins such as TNFRI, TNRII, and FcγRIII receptors.

In our study group, apart from anti-TNF treatment, other drugs were also used (mesalamine, corticosteroids or azathioprine), whose influence on the apoptosis process cannot be excluded, and which may affect the expression of individual receptors. However, in subgroups, patients did not differ statistically in mesalamine, corticosteroids and azathioprine treatment. Therefore, we did not perform an analysis in this respect. From the studies conducted, it is known that the use of azathioprine and anti-TNF drugs may be more effective than the use of biological drugs in monotherapy. In our case, however, trial treatment with azathioprine was used in every patient. Subject who did not continue thiopurine therapy showed intolerance to the drug. Azathioprine were applied for a period not shorter than 3 months before being included in biological treatment. All persons enrolled in the study were therefore on stable doses of the drugs. There are no reports in the literature on the influence of mesalamine, corticosteroids or azathioprine on the receptors which we studied (20). However, it can be speculated that due to the reduction in the inflammatory process, drugs such as corticosteroids and azathioprine may contribute to accelerating the elimination of inflammatory cells. This should have no effect on the expression of membrane proteins, disturbances to which result from genetic polymorphisms, and such a situation cannot be ruled out in our study group.

Regardless of the unexplained mechanism of anti-TNF action, in current clinical practice, there is a necessity to identify molecular mediators of inflammation, primarily in order to describe molecular markers of non-response and perhaps in the future, implement it in everyday clinical practice. A great scientific effort has been taken to identify useful and reliable indicators of non-response rate, but to date, not one of the genes analyzed is supported by strong evidence. *TNFRSF1B* appears to be an obvious candidate, considering that it codes for one of two TNFα receptors, namely TNF-RI. This gene is expressed on the surface of lymphocytes, epithelial cells, and macrophages (3). According to the literature, TNF-RI and TNF-RII receptors are identified as responsible for activating the apoptosis cascade. To some extent, functions of receptors may overlap, depending on the participation of different factors in signal transducing, the degree of cell activation and the environment. Moreover, TNF-RII activation might contribute to stimulate apoptosis, limiting immune response in autoreactive T cells (21, 22). Therefore, it seems that the decreased expression level of *TNFRSF1B* may be responsible for reduced patients' response by decreasing the ability of T cells elimination.

*FCGR3A* is a key player in the antibody-dependent cell cytotoxicity (ADCC) mechanism – one of the possible ways anti-TNF acts. The increased mRNA level in non-responding patients described in this paper may disturb ADCC activity and, as a consequence, eliminate cells expressing tmTNFα on their surface. Also, it is noteworthy that previously we found SNP in 3'-UTR of the gene which occurred with increased frequency in refractory patients. Additionally, this variant is placed in a potential seed region for miRNA particles. This suggests a possible regulation mechanism of the *FCGR3A* expression level. However, to confirm this, further functional studies should be performed (10). Moreover, the participation of anti-TNF mAbs in ADCC was indicated in research based on modified cell cultures, stably expressing tmTNFα (23, 24). *In vivo*, the impact of anti-TNF on T cells cytotoxicity remains uncertain (25, 26).

In the context of *FCGR3A*, the activity of *ADAM17* seems to be of value. A significant decrement of the mRNA level was observed in our group of non-responding patients compared to responders and controls. Other than controlling the balance between sTNFα and tmTNFα, this metalloproteinase is important for the activity of FcγRIII. ADAM 17 is a potential enzyme which inactivates the FcγRIII receptor (also called CD16) by a cleavage after cytotoxicity induction. It has been shown that after inhibition of the enzyme, FcγRIII activity was not decreased. ADAM 17 is also constitutively expressed on a surface of natural killer (NK) cells and other leucocytes (27–30). Additionally, inhibitors of this metalloproteinase are expected to be an effective treatment for oncological patients with decreased activity of FcγRIII, which is a potential cause of anti-TNF lack of activity (31). However, there is no data referring to CD in this matter. Based on the results presented in this study, we may hypothesize that down-regulated activity of *ADAM17* might influence the activity of *FCGR3A* but direct evidence was not provided and this requires further investigation.

Another candidate gene which may function as a molecular predictor of non-response is *IL1B*. Increased levels of this cytokine were described in the plasma of CD patients and other diseases triggered by autoimmunity. Also, up-regulated levels of the *IL1B* gene mRNA in the biopsies of refractory patients were described by transcriptomic studies (32, 33). We have confirmed these results in our study. Likewise, in the *FCGR3A* gene, we described polymorphisms in 3'-UTR, potentially affecting the miRNA binding site and additionally SNP in exonic sequence (10). If these variants affect the expression level of the gene, they should be investigated experimentally.

In IBD, the search for an ideal predictive marker of non-response in biological treatment has been ongoing for many years. A large number of different factors were identified as associated with resistance in patients, although the lack of reproducibility and predefined protocols for sample collection, small analyzed groups, and often contradictory results prevent the scientific community from drawing strong conclusions. According to West et al., oncostatin M seems to be a strong candidate for molecular marker, since the high pretreatment expression of *OSM* was associated with the failure of anti-TNF treatment (34). In pediatric patients, down-regulated *SMAD7* expression in whole blood cells prior and after 2 weeks of anti-TNF treatment was identified as associated with a lack of response. It is of utmost interest that the authors did not find any differences in the case of *TNFRSF1B*, and *OSM* (35). As mentioned, mostly this type of study is limited by the sample size. This is also the most restrictive factor. Another problem is the lack of standardized lab protocols, which might strongly affect RT-qPCR results and it should be equalized to improve the quality of results obtained in different laboratories.

In the set of genes analyzed, we revealed differences in expression between responders and non-responders, although biopsies were collected prior to anti-TNF treatment, and the ability to respond was assessed after 3 months of biological treatment. To investigate the impact of anti-TNF mAbs on the expression of our targets' level we decided to use cell cultures. We studied two aspects – whether the anti-TNF treatment would modify the expression level of genes of interest and whether mAbs are able to induce apoptosis in cells collected from patients.

Additionally, *TNFRSF1B, IL1B, FAS, FCGR3A*, and *ADAM17* are genes that are possibly involved in the multifactorial mechanism of anti-TNF action. One probable explanation of biological treatment efficacy is the induction of apoptosis of T lymphocytes, whose activity might be dysfunctional in refractory patients. Changes in the expression of a set of genes included in biopsies, characterized for non-responders, indicate their possible impact on the lack of response. However, in the conditions tested, we did not find significant differences in any of the three groups investigated between T cells population which were treated with anti-TNF mABs and the control culture. The results concerning the occurrence of apoptosis after anti-TNF treatment are also contradictory. Researchers from the Netherlands showed that infliximab induced apoptosis *in vitro* on lymphocytes isolated from *lamina propria*, as well as from PBMC by activation of caspase-3 in patients who responded to the treatment. However, it was not clear if this result was a consequence of direct activation through tmTNFα or a simple block of contact between tmTNFα and TNF receptors bearing cells (36, 37). Similar results were obtained in monocytes cultures from healthy individuals and responding patients (38). Other studies did not confirm these observations in monocytes (39) and lymphocytes (8) cultures. Although we did not show apoptosis induction after infliximab treatment, our results are strictly limited. First, we measured the overall level of apoptosis, not direct pathways or caspases activation. Secondly, the conditions used in the experiment are narrow, and possibly in wider circumstances, results might be different.

The impact of infliximab on apoptosis and its influence on the expression level of selected genes was measured. The mRNA level was compared with the culture treated with infliximab and parallel control in responders, non-responders, and healthy individuals. Three genes, *TNFRSF1B* and *FAS*, both of which are involved in apoptosis and *FCGR3A*, which is essential for the AICC mechanism, showed the same tendency. In the group of healthy individuals, infliximab induced significant up-regulation of genes, in responding patients no change was observed, and in the case of non-responders, the expression was down-regulated. *FAS* is coding for a key AICD death receptor and is involved in the process of autoreactive T cells elimination as well as *TNFRSF1A*. Meng et al. showed that *TNFRSF1B* might also participate in apoptosis control. In the activated T lymphocytes, isolated from PBMC of healthy individuals, apoptosis was induced. The silencing of *TNFRSF1B*, in contrast to *TNFRSF1A*, inhibited cell death. Similar results were obtained in Jurkat cells treated with an anti-TNF drug. What is of value is that stimulation of healthy T cells induced up-regulation of *FAS*, and *TNFRSF1B* expression. This is possibly one of the AICD mechanisms in which the antibody sensitizes cells to the influence of apoptotic signals by induction of death receptors expression on the cell surface (40). These results are in line with previous observations concerning the impact of TNF-RII stimulation on inhibition of T lymphocyte proliferation and cytokine expression (41). Schmitt et al. also identified that increased expression of *TNFRSF1B* in T cells from *lamina propria* prior to the treatment is associated with an increased response (9). The amount of evidence confirming the contribution of *TNFRSF1B* in apoptosis of T lymphocytes is increasing, which goes beyond its predominantly described function of proliferation stimulation. Here, we confirmed the observation that anti-TNF in healthy cells stimulates the up-regulation of *FAS* and *TNFRSF1B*. However, we additionally determined that the opposed effect occurred in cells obtained from non-responders. Presumably, due to molecular abnormalities, instead of activation of both genes, down-regulation is presented, which finally results as insensitivity to apoptotic signals. This is an interesting hypothesis, which should be verified by the scientific community.

In summary, our research contributes to understanding the mechanisms behind the lack of a primary response in CD patients. We have observed that in naïve non-responding patients, there is a changed expression of proteins involved in the process of apoptosis and elimination of pro-inflammatory cells at the mucosal tissue level. Perhaps the assessment of an appropriate panel of apoptotic genes will be a valuable future marker for the first therapy in IBD patients. However, more detailed research is needed in this area.

## Data Availability Statement

The raw data supporting the conclusions of this article will be made available by the authors, without undue reservation.

## Ethics Statement

The studies involving human participants were reviewed and approved by the Bioethical Committee of the University of Medical Sciences in Poznan, Poland, under Resolution No. 762/13 approved on 9 November 2013 and Resolution No. 1042/18 approved on 11 October 2018. All experiments were performed in accordance with the principles of the 1964 Declaration of Helsinki with its later amendments. The patients/participants provided their written informed consent to participate in this study. Written informed consent was obtained from the individual(s) for the publication of any potentially identifiable images or data included in this article.

## Author Contributions

LL-S and MS-Z: conceptualization. MW and LL-S: methodology and validation. MW, LL-S, JS-Z, KS-E, KW, PE, AW, and IK-K: investigation. MW: formal analysis. MW, LL-S, and MS-Z: writing-original draft preparation. PE and JS-Z: critical revision of the manuscript. KS-E, MS-Z, and RS: funding acquisition. RS and AD: supervision. All authors contributed to the article and approved the submitted version.

## Conflict of Interest

The authors declare that the research was conducted in the absence of any commercial or financial relationships that could be construed as a potential conflict of interest.
