# Supplementary material for: Effect of Anti-TNF Therapy on Mucosal Apoptosis Genes Expression in Crohn's Disease
Source: Front Immunol. 2021 Mar 9;12:615539. doi: 10.3389/fimmu.2021.615539 (PMC7985326; doi:10.3389/fimmu.2021.615539)
Supplement: Supplementary file 1 [file Data_Sheet_1.docx]

Supplementary Material

# Supplementary Data

**Table S1**. Mucosal expression of *ADAM17* mRNA in CD patients and controls

| Groups | Number | ∆Ct  (Median) | Q1-Q3 | *p*-value |
| --- | --- | --- | --- | --- |
| Responders noninflammed mucosa (RN) | 10 | -5.13 | -5.30; -4.75 | 0.4435 (RN *vs* C)  0.0110 (RN *vs* NH)  0.0081 (RN *vs* NI)  0.436 (RN *vs* RI) |
| Responders inflammed mucosa (RI) | 9 | -5.19 | -5.27; -4.88 | 0.4322 (RI *vs* C)  0.0155 (RI *vs* NH)  0.0053 (RI *vs* NI) |
| Nonresponders noninflammed mucosa (NN) | 6 | -7.10 | -7.65; -6.57 | 0.0121 (NN *vs* C)  0.4181 (NN *vs* NI) |
| Nonresponderes inflammed mucosa (NI) | 6 | -7.19 | -7.31; -7.08 | 0.004 (NI *vs* C) |
| Controls | 7 | -4.40 | -6.07; -3.85 | - |

Q1, first quartile; Q3; third quartile

**Table S2**. Mucosal expression of *FAS* mRNA in CD patients and controls

| Groups | Number | ∆Ct  (Median) | Q1-Q3 | *p*-value |
| --- | --- | --- | --- | --- |
| Responders noninflammed mucosa (RN) | 12 | -7.88 | -8.45; -7.29 | *ns* |
| Responders inflammed mucosa (RI) | 12 | -7.14 | -7.47; -6.85 | *ns* |
| Nonresponders noninflammed mucosa NN | 6 | -7.70 | -8.03; -7.49 | *ns* |
| Nonresponders inflammed mucosa (NI) | 6 | -6.83 | -7.16; -6.56 | *ns* |
| Controls | 10 | -6.58 | -7.74; -5.08 | - |

*ns*, not significant in on based on multiple comparison non-parametric Kruskal-Wallis test

**Table S3**. Mucosal expression of *FCGR3A* mRNA in CD patients and controls

| Groups | Number | ∆Ct  (Median) | Q1-Q3 | *p*-value |
| --- | --- | --- | --- | --- |
| Responders noninflammed  mucosa (RN) | 12 | -7.88 | -8.45; -7.29 | 0.2254 (RN *vs* C)  0.1463 (RN vs NN)  0.0151 (RN vs NI)  0.4294 (RN vs RI) |
| Responders inflammed  mucosa (RI) | 12 | -7.14 | -7.469; -6.85 | 0.2203 (RI *vs* C)  0.1517 (RI vs NH)  0.0250 (RI vs NI) |
| Nonresponders noninflammed  mucosa NN | 6 | -7.70 | -8.03; -7.49 | 0.3684 (NN *vs* C)  0.0030 (NN vs NI) |
| Nonresponders inflamed  mucosa (NI) | 6 | -6.83 | -7.16; -6.56 | 0.0044 (NI *vs* C) |
| Controls | 10 | -6.58 | -7.74; -5.08 | - |

**Table S4**. Mucosal expression of *IL1B* mRNA in CD patients and controls

| Groups | Number | ∆Ct  (Median) | Q1-Q3 | *p*-value |
| --- | --- | --- | --- | --- |
| Responders noninflammed mucosa (RN) | 8 | -5.47 | -6.43; -4.94 | 0.0393 (RN *vs* C)  0.4915 (RN vs NN)  0.0413 (RN vs NI)  0.4666 (RN vs RI) |
| Responders inflammed mucosa (RI) | 7 | -6.20 | -8.20; -3.91 | 0.0551 (RI *vs* C)  0.4700 (RI vs NN)  0.0530 (RI vs NI) |
| Nonresponders noninflammed mucosa NN | 6 | -5.44 | -7.47; -4.79 | 0.0412 (NN *vs* C)  0.0425 (NN vs NI) |
| Nonresponders inflammed mucosa (NI) | 6 | -3.41 | -3.634; -3.32 | 0.0002 (NI *vs* C) |
| Controls | 8 | -8.63 | -9.09; -7.45 | - |

**Table S5**. Mucosal expression of *IL1R* mRNA in CD patients and controls

| Groups | Number | ∆Ct  (Median) | Q1-Q3 | *p*-value |
| --- | --- | --- | --- | --- |
| Responders noninflammed mucosa (RN) | 11 | -5.76 | -6.07; -5.27 | 0.0499 (RN *vs* C)  0.2713 (RN vs NN)  0.3997 (RN vs NI)  0.2380 (RN vs RI) |
| Responders inflammed mucosa (RI) | 11 | -5.38 | -5.67; -4.50 | 0.0047 (RI *vs* C)  0.1298 (RI vs NN)  0.2890 (RI vs NI) |
| Nonresponders noninflammed mucosa NN | 6 | -7.26 | -7.46; -5.28 | 0.2474 (NN *vs* C)  0.2592 (NN vs NI) |
| Nonresponders inflammed mucosa (NI) | 6 | -5.25 | -6.16; -4.63 | 0.0417 (NI *vs* C) |
| Controls | 8 | -6.99 | -7.65; -6.39 | - |

**Table S6**. Mucosal expression of *TNFRSF1B* mRNA in CD patients and controls

| Groups | Number | ∆Ct  (Median) | Q1-Q3 | *p*-value |
| --- | --- | --- | --- | --- |
| Responders noninflammed  mucosa (RN) | 11 | -6.68 | -7.04; -6.12 | 0.0967 (RN *vs* C)  0.0015 (RN vs NN)  0.0308 (RN vs NI)  0.0619 (RN vs RI) |
| Responders inflammed  mucosa (RI) | 12 | -5.53 | -6.40; -4.96 | 0.4944 (RI *vs* C)  0.0007 (RI vs NN)  0.0010 (RI vs NI) |
| Nonresponders noninflammed mucosa NN | 6 | -11.38 | -11.68; -10.83 | 0.0015 (NN *vs* C)  0.4498 (NN vs NI) |
| Nonresponders inflammed  mucosa (NI) | 6 | -10.06 | -10.48; -9.44 | 0.0025 (NI *vs* C) |
| Controls | 6 | -5.65 | -5.81; -5.39 | - |

**Table S7.** PBMC expression of *ADAM17* mRNA in CD patients and controls

| Groups | Anti-TNF mAbs  [72 h] | Number | ∆Ct  (Median) | Q1-Q3 | *p*-value |
| --- | --- | --- | --- | --- | --- |
| Responders cell culture | + | 6 | -8.80 | -9.13; -8.00 | 0.8182 |
|  | - | 6 | -8.82 | -9.12; -8.19 |  |
| Nonresponders cell culture | + | 6 | -8.85 | -10.81; -8.54 | 1.000 |
|  | - | 6 | -9.19 | -9.35; -8.88 |  |
| Controls cells culture | + | 6 | -7.17 | -7.27; -7.04 | 0.0486 |
|  | - | 8 | -8.24 | -8.72; -8.00 |  |

mAbs, monoclonal antibody

**Table S8**. PBMC expression of *FAS* mRNA in CD patients and controls.

| Groups | Anti-TNF mAbs  [72 h] | Number | ∆Ct  (Median) | Q1-Q3 | *p*-value |
| --- | --- | --- | --- | --- | --- |
| Responders cell culture | + | 6 | -8.30 | -10.40; -7.50 | 0.8182 |
|  | - | 6 | -8.65 | -9.43; -8.56 |  |
| Nonresponders cell culture | + | 6 | -11.18 | -11.22; -11.11 | 0.0087 |
|  | - | 6 | -10.32 | -10.66; -9.68 |  |
| Controls cells culture | + | 6 | -7.50 | -8.30; -7.19 | 0.0200 |
|  | - | 8 | -8.86 | -9.48; -8.30 |  |

**Table S9**. PBMC expression of *FCGR3A* mRNA in CD patients and controls.

| Groups | Anti-TNF mAbs  [72 h] | Number | ∆Ct  (Median) | Q1-Q3 | *p*-value |
| --- | --- | --- | --- | --- | --- |
| Responders cell culture | + | 6 | -10.45 | -10.70; -8.27 | 0.8182 |
|  | - | 6 | -9.63 | -10.18; -9.15 |  |
| Nonresponders cell culture | + | 6 | -12.22 | -12.66; -11.72 | 0.0087 |
|  | - | 6 | -9.15 | -9.36; -8.96 |  |
| Controls cells culture | + | 6 | -6.34 | -7.50; -5.44 | 0.0012 |
|  | - | 7 | -9.34 | -9.69; -8.81 |  |

**Table S10.** PBMC expression of *IL1B* mRNA in CD patients and controls.

| Groups | Anti-TNF mAbs  [72 h] | Number | ∆Ct  (Median) | Q1-Q3 | *p*-value |
| --- | --- | --- | --- | --- | --- |
| Responders cell culture | + | 6 | -14.10 | -15.17; -12.46 | 0.1797 |
|  | - | 6 | -11.95 | -12.32; -11.79 |  |
| Nonresponders cell culture | + | 6 | -14.42 | -14.84; -14.29 | 0.6623 |
|  | - | 6 | -14.08 | -14.55; -13.29 |  |
| Controls cells culture | + | 6 | -8.24 | -8.41; -8.06 | 0.0022 |
|  | - | 6 | -10.70 | -11.42; -10.05 |  |

**Table S11**. PBMC expression of *IL1R* mRNA in CD patients and controls.

| Groups | Anti-TNF mAbs  [72 h] | Number | ∆Ct  (Median) | Q1-Q3 | *p*-value |
| --- | --- | --- | --- | --- | --- |
| Responders cell culture | + | 6 | -13.70 | -13.91; -13.15 | 0.8182 |
|  | - | 6 | -13.73 | -13.96; -13.55 |  |
| Nonresponders cell culture | + | 6 | -15.05 | -15.09; -14.37 | 0.0573 |
|  | - | 6 | -13.62 | -14.00; -13.35 |  |
| Controls cells culture | + | 6 | -10.23 | -11.97; -9.82 | 0.1320 |
|  | - | 6 | -11.06 | -12.90; -10.73 |  |

**Table S12**. PBMC expression of *TNFRSF1B* mRNA in CD patients and controls.

| Groups | Anti-TNF mAbs  [72 h] | Number | ∆Ct  (Median) | Q1-Q3 | *p*-value |
| --- | --- | --- | --- | --- | --- |
| Responders cell culture | + | 6 | -8.56 | -9.06; -8.51 | 0.8182 |
|  | - | 6 | -8.63 | -8.64; -8.49 |  |
| Nonresponders cell culture | + | 6 | -10.15 | -12.14; -9.88 | 0.0095 |
|  | - | 6 | -9.09 | -9.27; -8.68 |  |
| Controls cells culture | + | 6 | -5.60 | -5.69; -5.33 | 0.0007 |
|  | - | 8 | -9.30 | -9.79; -8.81 |  |
